# Bispecific Antibodies—A New Hope for Patients with Diffuse Large B-Cell Lymphoma

**DOI:** 10.3390/jcm14155534

**Published:** 2025-08-06

**Authors:** Romeo Gabriel Mihaila, Samuel B. Todor

**Affiliations:** Faculty of Medicine, “Lucian Blaga” University of Sibiu, 550024 Sibiu, Romania; romeo.mihaila@ulbsibiu.ro

**Keywords:** bispecific antibodies, blinatumomab, CAR T-cell, epcoritamab, glofitamab, mosunetuzumab, T-cell-engaging antibodies

## Abstract

T-cell-engaging antibodies are a promising new type of treatment for patients with refractory or relapsed (R/R) diffuse large B-cell lymphoma, which has changed the prognosis and evolution of these patients in clinical trials. Bispecific antibodies (BsAbs) bind to two different targets (B and T lymphocytes) at the same time and in this way mimic the action of CAR (chimeric antigen receptor) T-cells. They are the T-cell-engaging antibodies most used in practice and are a solution for patients who do not respond to second- or later-line therapies, including chemoimmunotherapy, followed by salvage chemotherapy and hematopoietic stem cell transplantation. They are a therapeutic option for patients who are ineligible for CAR T-cell therapy and are also active in those with prior exposure to CAR T-cell treatment. A remarkable advantage of BsAbs is their rapid availability, even if the disease progresses rapidly, unlike CAR T-cell treatment, and they avoid the practical and financial challenges raised by autologous CAR T-cell therapies. CAR-T has been proven to have better efficacy compared to BsAbs, but cytokine release syndrome and neurotoxicity have appeared significantly more frequently in patients treated with CAR T-cells. The possibility of combining BsAbs with chemotherapy and their administration for relapses or as a frontline therapy is being studied to increase their efficacy. BsAbs are a life-saving therapy for many patients with diffuse large B-cell malignant non-Hodgkin’s lymphoma (NHL) who have a poor prognosis with classical therapies, but are not without adverse effects and require careful monitoring.

## 1. Introduction

Diffuse large B-cell lymphoma (DLBCL) is the most common type of lymphoma, yet it remains a heterogeneous B-cell malignancy [1]. Traditional chemotherapy faces several limitations, including treatment resistance, cumulative toxicities, and the depletion of hematopoietic reserves [2]. Despite significant therapeutic advances, approximately 30–40% of patients experience relapse or refractory disease, which continues to be associated with a poor prognosis [3,4]. For decades, fit patients with R/R DLBCL were treated with high-dose chemotherapy followed by autologous hematopoietic stem cell transplantation (auto-HSCT). However, the outcomes are unfavorable for those who fail to respond to salvage therapy or are ineligible for a transplant [5].

The treatment landscape of DLBCL has undergone a significant transformation with the introduction of targeted therapies, such as monoclonal antibodies, antibody–drug conjugates (ADCs), and adoptive T-cell therapies [6].

Among these, rituximab has stood the test of time since its late 1990s introduction and remains a cornerstone of DLBCL therapy [2,6]. In a cohort of 403 patients treated with R-CHOP, the 5-year overall survival rate (OSR) reached 66.5%, and the 2-year progression-free survival (PFS) was 68%. However, 34.4% experienced relapse and 22.6% were refractory. After salvage therapy, the median OS and PFS dropped to just 6.7 and 5.1 months, respectively [7].

Despite advances in frontline therapy, a significant subset of patients with DLBCL relapse within two years and face poor outcomes with standard salvage approaches. Prognostic indicators, such as the POD24 (progression of disease within 24 months), a low LMR (lymphocyte–monocyte ratio), and elevated C-reactive protein at the time of relapse, have been associated with resistance to treatment and inferior survival. These limitations highlight the urgent need for more effective and accessible therapeutic options [8].

What therapeutic strategies could improve outcomes? Advances include the development of more potent monoclonal antibodies, CAR T-cells, BsAbs, ADCs, immune checkpoint inhibitors, and small molecules targeting intracellular signaling pathways [2].

One such agent, polatuzumab vedotin, a CD79b-targeting ADC, has shown improved outcomes when combined with chemotherapy and rituximab for the first-line treatment of high-risk patients [6].

CAR T-cell therapy is now employed as a second-line treatment in patients refractory to first-line chemoimmunotherapy [1]. In fact, anti-CD19 CAR T-cell therapy has become the preferred option for high-risk DLBCL, specifically those with primary refractory disease or relapse within 12 months of the first-line treatment [9]. The FDA approved Axicabtagene ciloleucel and Lisocabtagene maraleucel for this indication in 2022 and 2023, respectively [10].

These therapies have achieved complete response rates between 40 and 58% [11] and significantly improved the progression-free survival compared to traditional salvage regimens. Moreover, they offer acceptable safety and high efficacy, even among elderly or comorbid patients [10]. However, up to 50% of patients may still relapse following CAR T-cell therapy [12].

The purpose of this review is to provide an updated overview of the clinical utility and safety profile of BsAbs in DLBCL, with particular attention to their use in R/R settings. Additionally, we compare BsAbs therapies with CAR T-cell approaches, in terms of its efficacy, accessibility, toxicity, and patient eligibility.

For this review, we conducted a comprehensive literature search of three major databases: PubMed, Embase, and Web of Science. The aim of the search was to identify studies assessing the efficacy, safety, and clinical utility of bispecific antibodies (BsAbs) in DLBCL, as well as studies comparing BsAbs with CAR T-cell therapies in the context of R/R disease.

The search strategy employed a combination of MeSH terms and free-text keywords, including but not limited to “Diffuse large B-cell lymphoma”, “DLBCL”, “bispecific antibodies”, “BsAbs”, “CAR T-cell”, “chimeric antigen receptor T-cell”, “relapsed”, “refractory”, “immunotherapy”, “safety”, “toxicity”, and “efficacy.” Boolean operators were used to enhance the search sensitivity and specificity.

Only articles involving adult patients (≥18 years) were considered. The eligible studies included clinical trials, prospective or retrospective observational studies, real-world evidence reports, and relevant systematic reviews or meta-analyses. Studies were included if they reported outcomes such as the overall response rate, complete response rate, progression-free survival, overall survival, toxicity profiles, or clinical eligibility criteria related to BsAbs or CAR T-cell therapies in DLBCL. Studies focused solely on preclinical models, pediatric populations, or those with insufficient outcome data were excluded.

## 2. Pharmacodynamics of Bispecific Antibodies

BsAbs are a novel class of therapy. They bind to two different targets (B and T lymphocytes) at the same time and in this way mimic the action of CAR T-cells [1]. BsAbs are a promising treatment option for patients with relapsed DLBCL after the failure of second- or later-line therapies and have demonstrated that they are effective even in a subset of patients who relapsed after treatment with CD19 CAR T-cells [10].

Most antibodies produced by the human immune system are naturally bivalent and monospecific, meaning they possess two identical binding sites that recognize a single antigen target. In contrast, BsAbs are bioengineered molecules capable of simultaneously engaging two distinct epitopes, typically one on a tumor cell and another on an immune effector cell. This dual-targeting capability enables BsAbs to bridge immune cells—such as T-cells or natural killer cells—with malignant cells, thereby promoting immune-mediated cytotoxicity [11].

**Figure 1:**
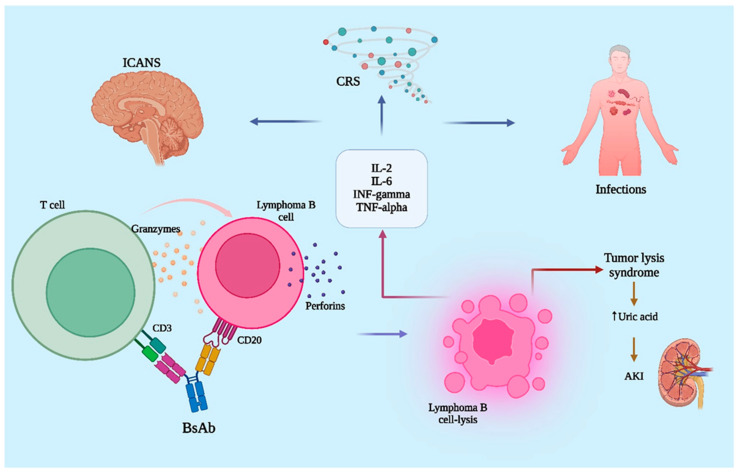
Mechanism of action of bispecific antibodies (BsAbs) in lymphoma and associated adverse effects. BsAbs simultaneously bind to CD3 on T-cells and CD20 on lymphoma cells, facilitating targeted T-cell-mediated cytotoxicity. AKI, acute kidney injury; BsAb, bispecific antibody; CD3, cluster of differentiation 3; CD20, cluster of differentiation 20; CRS, cytokine release syndrome; ICANS, immune effector cell-associated neurotoxicity syndrome; IFN-γ, interferon gamma; IL-2, interleukin-2; IL-6, interleukin-6; TNF-α, tumor necrosis factor alpha; TLS, tumor lysis syndrome. Created in Biorender. Samuel B. Todor (2025) https://app.biorender.com/illustrations/6862abe589408258b34db174.

The destruction of tumor cells is primarily driven by antibody-dependent cellular cytotoxicity (ADCC), a mechanism involving the release of cytolytic enzymes like perforins and granzymes. As a consequence of this immune activation, the tumor microenvironment becomes enriched with pro-inflammatory cytokines, such as interleukin-2 (IL-2), interleukin-6 (IL-6), interferon gamma (IFN-γ), and tumor necrosis factor alpha (TNF-α). While these cytokines are critical for mounting an effective antitumor response, their systemic release may lead to significant inflammatory toxicity. The principal adverse effects linked to BsAbs therapy include infectious complications, the unintended targeting of non-malignant tissues (on-target off-tumor toxicity), cytokine release syndrome (CRS), immune effector cell-associated neurotoxicity syndrome (ICANS), and tumor lysis syndrome (TLS) (Figure 1) [13].

## 3. Bispecific Antibodies Products

Several bispecific antibody agents have entered clinical use, most notably epcoritamab and glofitamab, both of which received FDA approval in 2023 for the treatment of relapsed or refractory DLBCL after at least two prior lines of therapy, including cases of DLBCL not otherwise specified and those transformed from indolent lymphomas [3,6,14,15]. These agents are indicated for patients who have either relapsed after or are ineligible for CAR-19 therapy, and they have demonstrated efficacy even in those previously treated with CAR T-cells [3,14]. Epcoritamab additionally received regulatory approval in Japan in September 2023 due to favorable results in terms of both its effectiveness and safety in clinical studies [16].

BsAbs have been shown to induce overall response rates (ORRs) greater than 50%, with many patients maintaining remission beyond two years. While epcoritamab is administered subcutaneously and glofitamab intravenously, it is the predictable timing and manageable severity of CRS and ICANS with these agents, combined with appropriate outpatient monitoring strategies, that enable their use in outpatient settings [12]. The reported complete response (CR) rates typically range from 30% to 40% in R/R settings [17].

Epcoritamab is a humanized IgG1 antibody designed to engage CD3 on T-cells and CD20 on malignant B-cells, facilitating T-cell-driven cytotoxicity. The preclinical findings in cynomolgus monkeys showed lower cytokine peaks following subcutaneous injection compared to intravenous delivery, supporting the clinical adoption of the subcutaneous route to mitigate CRS [16,18]. In this study involving 157 patients with R/R large B-cell lymphoma, including 61.1% with primary refractory disease and 38.9% with prior CAR T-cell therapy, the treatment yielded promising outcomes. At a median follow-up of 10.7 months, the overall response rate was 63.1%, with a complete response rate of 38.9%. The median duration of response was 12 months, and among the complete responders, the median had not yet been reached [19].

In the EPCORE NHL-2 phase 1b/2 trial, epcoritamab plus GemOx showed high efficacy in 103 transplant-ineligible patients with R/R DLBCL, achieving an ORR of 85% and CR rate of 61%. With a median follow-up of 13.2 months, the median CR duration was 23.6 months and the OS was 21.6 months, demonstrating a durable benefit in a heavily pretreated population [20].

In a Phase I trial of glofitamab, 171 patients with R/R B-cell non-Hodgkin lymphoma (B-NHL) were treated, most of whom were heavily pretreated and refractory to prior therapies. Among them, 127 patients had aggressive histologies, including DLBCL. The overall response rate was 53.8%, with a CR rate of 36.8%. At the recommended Phase II dose, the response improved to a 65.7% ORR and 57.1% CR. Notably, 84.1% of the patients who achieved a CR maintained it during a follow-up period of up to 27.4 months, confirming its strong and durable activity, particularly in DLBCL [21].

Among the first reports evaluating glofitamab in R/R DLBCL, this study demonstrated a 39% complete response rate among 154 treated patients, including a 35% CR in those previously exposed to CAR T-cell therapy. The responses were typically rapid, with a median time to CR of 42 days, and 78% of the CRs were ongoing at 12 months. The 12-month progression-free survival reached 37%, highlighting its early and durable efficacy [22].

When evaluating combinatory therapy, the phase 3 STARGLO trial demonstrated that Glofit-GemOx used as a second-line or later therapy significantly improved the overall survival compared to R-GemOx in transplant-ineligible patients with R/R DLBCL. At a median follow-up of 20.7 months, the median OS reached 25.5 months versus 12.9 months (HR 0.62), supporting Glofit-GemOx as a more effective therapeutic option in this population [23].

Another agent, blinatumomab, a CD3/CD19 bispecific T-cell engager, recruits cytotoxic T-cells to target CD19-positive malignant cells [24]. The early reports of blinatumomab’s utility in post-HSCT consolidation indicate it is a safe and promising approach for high-risk B-cell lymphomas. In a pilot study of 14 patients with relapsed DLBCL or transformed follicular lymphoma who underwent an autologous stem cell transplant (auto-SCT) with BEAM conditioning, one cycle of blinatumomab consolidation starting day 42 post-transplant was well tolerated with no adverse events reported. At 100 days post-auto-SCT, 86% achieved complete remission, and at 1 year, 50% remained in remission. The patients who relapsed had lower pretreatment CD8:CD4 T-cell ratios [24].

Another phase 2 trial of blinatumomab in heavily pretreated R/R DLBCL (median of three prior therapies) showed a 43% overall response rate after one cycle, including 19% with complete responses. Stepwise dosing (9-28-112 μg/d) was better tolerated than flat dosing (112 μg/d), with the common adverse events including tremor, fever, and fatigue. Grade 3 neurologic events led to treatment discontinuation in 22% of patients, which mostly resolved afterward. The flat-dose cohort was halted due to severe neurotoxicity [25]. In Richter transformation, adding blinatumomab consolidation after R-CHOP in patients without a complete response showed encouraging results. Among 25 patients treated with blinatumomab induction, 20% achieved complete remission and 16% a partial response. The overall response rate was 46%, with a 36% complete remission rate in the full cohort [26].

Mosunetuzumab, a CD20/CD3 BsAb [27], showed durable efficacy in R/R B-NHL—including DLBCL and transformed follicular lymphoma—with no unexpected safety concerns. In 197 patients at higher doses, the common adverse events included mostly low-grade cytokine release syndrome, neutropenia, hypophosphatemia, fatigue, and diarrhea. The overall response rates were 34.9% in aggressive B-NHL and 66.2% in indolent B-NHL, with complete responses of 19.4% and 48.5%, respectively. The median duration of the complete response exceeded 20 months, confirming mosunetuzumab’s effective and manageable safety profile [28]. In R/R LBCL (including DLBCL), mosunetuzumab plus polatuzumab vedotin achieved a 59.2% overall and 45.9% complete response rate. The median progression-free survival was 11.4 months and the overall survival was 23.3 months. Grade ≥3 neutropenia occurred in 25% of the cases, and cytokine release syndrome in 16.7%. The regimen showed durable efficacy and manageable safety as a second-line therapy [29].

However, in a small phase 2 trial comparing mosunetuzumab plus Pola-CHP (Pola-M-CHP) to Pola-R-CHP for first-line DLBCL, the complete response rates were similar (72.5% vs. 77.3%). The 24-month progression-free survival was 70.8% with Pola-M-CHP versus 81.8% with Pola-R-CHP. The pharmacodynamics confirmed mosunetuzumab’s activity, but no clinical benefit over Pola-R-CHP was observed [30].

After prior CAR T-cell therapy, mosunetuzumab showed better responses when administered later, with the responders exhibiting higher lymphocyte counts and increased CD4+, CD8+, and activated CD8+ T-cells after treatment. The nonresponders showed decreased CAR transgene levels. This first study of immune and CAR transgene dynamics post-BsAb highlights the impact of timing on mosunetuzumab’s efficacy after CAR-T [31].

Long-term remissions have been observed following treatment with epcoritamab or glofitamab in many cases of B-cell non-Hodgkin lymphoma (B-NHL), with the response durability appearing comparable between the two agents. Although some studies have attempted to assess the optimal treatment duration, no definitive consensus currently exists, and further research is needed to clarify the best strategies for therapy length and long-term management [3,4].

The emerging data from randomized trials support the use of BsAbs not only as a monotherapy but also in combination with established chemoimmunotherapy regimens [3]. This growing therapeutic interest is reflected in the volume of ongoing research: in the United States alone, 62 clinical trials are underway exploring CAR T-cell therapies and 13 are focused on BsAbs, encompassing over 6200 patients enrolled or projected to enroll [32].

A detailed overview of the BsAbs-related clinical trials is presented in Table 1.

## 4. Comparison of CAR T-Cell Therapy and BsAbs

**Figure 2:**
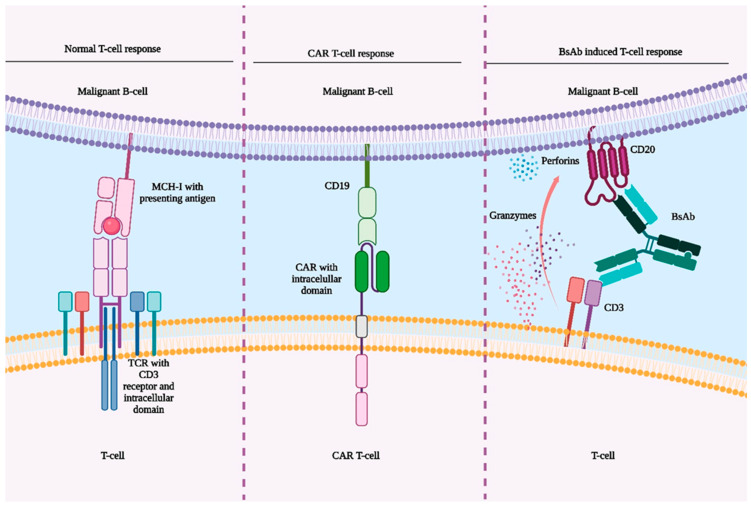
Mechanisms of T-cell activation against malignant B-cells. *Left:* Normal T-cell response via MHC-I antigen presentation to the TCR/CD3 complex. *Middle:* CAR T-cell therapy bypasses MHC-I by recognizing CD19 through a synthetic receptor with intracellular signaling domains. *Right:* Bispecific antibodies (BsAbs) simultaneously bind to CD3 on T-cells and CD20 on B-cells, redirecting T-cell cytotoxicity via perforin and granzyme release. Abbreviations: BsAb, bispecific antibody; CAR, chimeric antigen receptor; CD3, cluster of differentiation 3; CD19, cluster of differentiation 19; CD20, cluster of differentiation 20; MCH-I, major histocompatibility complex class I; TCR, T-cell receptor. Created in Biorender. Samuel B. Todor (2025) https://app.biorender.com/illustrations/6864f9f2d1fadb4f5400e863.

Chimeric antigen receptors (CARs) are engineered receptors that redirect the specificity and function of T lymphocytes or other immune cells toward the target antigens in a single synthetic construct. In cancer immunotherapy, CARs are designed to recognize tumor-associated antigens, enabling the generation of large numbers of uniformly targeted T-cells. Infusing these tumor-specific CAR T-cells circumvents the limitations of traditional active immunization. Unlike passive immunotherapy with monoclonal antibodies, CAR T-cells function as active agents with supraphysiologic potency, engaging tumor antigens to elicit both rapid and durable antitumor responses (Figure 2) [33,34].

The first CD19-directed CAR T-cell therapy approved by the U.S. Food and Drug Administration (FDA) was tisagenlecleucel (tisa-cel), initially for pediatric and young adult patients with acute lymphoblastic leukemia (ALL) based on the ELIANA trial, and subsequently for diffuse large B-cell lymphoma (DLBCL), high-grade B-cell lymphoma, and DLBCL transformed from follicular lymphoma as demonstrated in the JULIET trial [35,36,37]. Around the same period, axicabtagene ciloleucel (axi-cel) received approval for relapsed/refractory (R/R) diffuse large B-cell lymphoma (DLBCL) based on the results from the ZUMA-1 trial, with later approval extended to include R/R follicular lymphoma [38,39].

The optimal sequencing of the therapeutic strategies involving bispecific antibodies (BsAbs) and antibody–drug conjugates (ADCs) remains unclear, particularly regarding their impact on subsequent CAR T-cell therapy [40]. Both CAR T-cell therapy and BsAbs have emerged as important options for patients with relapsed/refractory (R/R) DLBCL, with expanding efficacy profiles as clinical experience and real-world data accumulate. A shared limitation of both modalities is the risk of cytokine release syndrome (CRS) [41], and neither approach consistently yields durable remission across all patient subsets [42]. While BsAbs, such as mosunetuzumab, are being explored in earlier lines of therapy, a study evaluating mosunetuzumab combined with Pola-CHP in a frontline setting did not demonstrate improved outcomes compared to standard Pola-R-CHP, though this likely reflects the strength of the comparator arm rather than a failure of the BsAbs approach [30]. These challenges are compounded by the fact that most of the available data come from heavily pretreated or refractory populations, including patients with prior exposure to CAR T-cell therapy or hematopoietic stem cell transplantation (HSCT), limiting the ability to define an evidence-based sequencing algorithm [24,31].

An increasing number of patients with DLBCL receive CD19 CAR T-cell therapy as a second-line treatment, particularly in cases of early relapse or primary refractory disease following initial chemoimmunotherapy. The clinical outcomes of this approach are influenced by both patient- and disease-specific factors, as well as prior treatment exposure [43]. The long-term data suggest that CD19 CAR T-cell therapy can lead to a cure in approximately 30–40% of patients with DLBCL and related aggressive B-NHLs, including those with high-risk features, such as primary refractory disease or relapse within one year of frontline therapy [14]. Additionally, other studies have reported that CD19 CAR T-cell therapy can induce durable complete responses in nearly half of patients with R/R DLBCL [40]. In a phase 2 trial, anti-CD19 CAR T-cell therapy combined with prophylactic anakinra achieved an ORR of 77% and a CR rate of 65%. The ICANS incidence was low (all grade, 19%, grade ≥ 3: 9.7%) with no grade 4–5 events, supporting prior preclinical evidence of anakinra’s neuroprotective effect [44].

A meta-analysis evaluating the efficacy and safety of CAR T-cell therapy and BsAbs in 1347 patients with R/R DLBCL demonstrated a significantly higher CR rate in those treated with CAR T-cells (0.51) compared to BsAbs (0.36; *p* < 0.01). This advantage remained consistent even after adjusting for the proportion of double-hit lymphoma. Similarly, the one-year progression-free survival was superior with CAR T-cell therapy (0.44) versus BsAbs (0.32; *p* < 0.01) [45]. Despite this efficacy, CAR T-cell therapy entails considerable logistical and financial challenges, including a manufacturing period of 3–4 weeks, the need for certified tertiary care centers, and per-patient costs similar to those of allogeneic bone marrow transplantation [14].

In this context, the ELM-1 post-CAR-T expansion cohort evaluated odronextamab—a CD20 × CD3 BsAb—in 60 patients with R/R DLBCL who had progressed after CAR-T. With a median follow-up of 16.2 months, the objective response rate was 48.3%, including 31.7% with complete responses. The median PFS and OS were 4.8 and 10.2 months, respectively, with responses consistent regardless of receiving a prior CAR-T product or relapse timing [46]. A similar pattern has been observed in other studies, such as with blinatumomab post-HSCT and Mosunetuzumab post-CAR T-cell therapy, suggesting that the timing of BsAbs administration may critically influence the outcomes [24,31]. It is possible that BsAbs exert a greater therapeutic impact when used after prior immune conditioning, such as CAR-T or HSCT. In contrast, trials investigating BsAbs in a frontline setting, such as Mosunetuzumab combined with Pola-CHP, have not shown significant superiority over standard regimens [30].

## 5. Advantages and Limits of Bispecific Antibodies

Historically, there have been limited therapeutic options for patients with R/R DLBCL who were ineligible for CAR T-cell therapy. BsAbs have emerged as a viable treatment alternative for this population, demonstrating promising efficacy and manageable safety profiles [9].

A major advantage of BsAbs is their rapid availability [3,4], which makes them particularly suitable for patients with rapidly progressing DLBCL [4], bypassing the production delays and logistical hurdles associated with autologous CAR T-cell therapies [3]. Moreover, although BsAbs share similar indications with CAR T-cells, they generally produce less severe immune-related adverse events and can be administered in standard hematology units without requiring individualized manufacturing processes [14].

Agents such as epcoritamab and glofitamab have demonstrated activity even in patients previously treated with CAR T-cell therapy [3]. Notably, multiomic analyses of the DLBCL tumor microenvironment have revealed that immune gene expression profiles and cell-of-origin classifications are significantly correlated with the clinical response to mosunetuzumab, but not to CD19-directed CAR T-cell therapies [42], indicating a potentially distinct pattern of responsiveness based on the immune milieu.

Furthermore, in Richter syndrome, a meta-analysis of 509 patients across 30 studies reported that BsAbs—either as monotherapy or in combination with chemotherapy debulking—achieved higher response rates than Bruton tyrosine kinase inhibitors [47]. Despite these encouraging results, there is currently no evidence of a cure with BsAbs monotherapy, likely due to limited follow-up. Ongoing studies are evaluating their integration with chemotherapy and their potential role in relapsed or even frontline settings [14].

Unfortunately, patients lacking CD20 expression cannot benefit from any of the four available BsAbs, highlighting the need for alternative therapeutic targets and novel agents to mitigate the risk of CD20 loss [48].

Blinatumomab, a CD19 × CD3 bispecific T-cell engager already approved for B-cell acute lymphoblastic leukemia (B-ALL), has also shown activity in certain B-cell non-Hodgkin lymphoma subtypes. However, its clinical use has been limited by a short half-life and the need for continuous intravenous infusion [49]. More recently, AZD0486, a next-generation CD19×CD3 bispecific antibody, has demonstrated encouraging efficacy in patients with heavily pretreated diffuse large B-cell lymphoma (DLBCL). The early data indicate a dose-dependent response, with activity observed at doses up to 15 mg. At therapeutic doses, AZD0486 is generally well tolerated, and step-up dosing (2SUD) helps mitigate the risk of cytokine release syndrome (CRS) and immune effector cell-associated neurotoxicity syndrome (ICANS). These findings support the potential of CD19-directed bispecifics to broaden the treatment options, especially for patients with CD20-negative or refractory disease [50].

The optimal sequencing of treatment lines in R/R DLBCL remains undefined, and the increasing number of available options makes clinical decision-making increasingly complex [43].

## 6. The Safety of Bispecific Antibodies

BsAbs exhibit a safety profile that is inherently linked to their mechanism of action [3,4]. The toxicity associated with BsAbs is generally manageable and comparable to that observed with other T-cell redirecting therapies [17]. BsAbs can trigger a robust systemic cytokine release, which may progress to CRS [51]. The most commonly reported adverse events associated with BsAb therapies are CRS and immune-mediated cytopenias [10]. Patients receiving BsAbs are prone to hematologic toxicities—anemia, thrombocytopenia, and neutropenia—potentially due to pro-inflammatory cytokine release or impaired hematopoiesis [52,53]. Supportive care, including blood product transfusions and G-CSF, may help correct cytopenias and lower infection risk.

The potential adverse events (AEs) commonly associated with CAR T-cell therapy, including ICANS, are infrequent in patients treated with BsAbs [27,54,55,56]. Neurological AEs, such as aphasia and tremor, were each reported in 7% of patients [57]. Conversely, aphasia was not seen with BsAbs, and tremor occurred as a rare incident with a low grade. CRS associated with BsAbs is manageable [57].

CRS remains the principal management challenge associated with BsAbs therapy [3]. Its severity can be mitigated through strategies such as step-up dosing, glucocorticoid premedication, and the use of subcutaneous administration routes [17]. Notably, dexamethasone and TNFα inhibitors have been shown to effectively reduce cytokine release, although they may partially impair antitumor activity. In contrast, IL-6 receptor blockade, inflammasome inhibition, and IL-1 receptor blockade preserve BsAbs efficacy but are less effective in controlling cytokine release [51]. For glofitamab, pretreatment with obinutuzumab has been shown to reduce adverse effects, while step-up dosing, a strategy commonly used across multiple bispecific antibodies, further helps mitigate toxicity [4]. Premedication with dexamethasone (Dex) was associated with a lower incidence and severity of CRS compared to other corticosteroids. CRS occurred in 48.5% of patients in the Dex group versus 73.2% in the non-Dex group, with fewer grade ≥3 and serious CRS events reported. The use of tocilizumab and corticosteroids to manage CRS was also lower in the Dex group [58].

Adverse events of grade ≥3 in patients treated with BsAbs have been reported with incidences of 2% for CRS, 1% for neurotoxicity, and 10% for infections, according to a meta-analysis [45]. In the Japanese EPCORE NHL-3 phase I/II trial, CRS occurrence was predictable, and the majority of adverse events were mild, with none necessitating treatment discontinuation [59]. ICANS is very rare and occurs significantly less frequently compared to patients receiving CD19-directed CAR T-cell therapy [3,17].

A retrospective single-center study reported that 50% of DLBCL patients treated with an anti-IL6/IL6R agent developed infections [41]. Infections remain a significant concern with BsAbs due to their potential to impair both B-cell and T-cell function and to induce lymphopenia. Clinical trials have documented infectious complications, including febrile neutropenia (in less than 5% of cases), urinary tract infections, pneumonia, and COVID-19 [58,60,61]. The elevated risk of bacterial, viral, and opportunistic infections associated with BsAbs therapy is primarily linked to T-cell exhaustion, neutropenia, and hypogammaglobulinemia induced by the treatment. This vulnerability stems from both direct T-cell activation and on-target off-tumor effects, where BsAbs targets are also expressed on healthy cells. For instance, CD19- and CD20-directed BsAbs can lead to B-cell depletion, while BsAbs targeting the B-cell maturation antigen (BCMA) may result in the elimination of normal plasma cells and antibodies [62].

**Figure 3:**
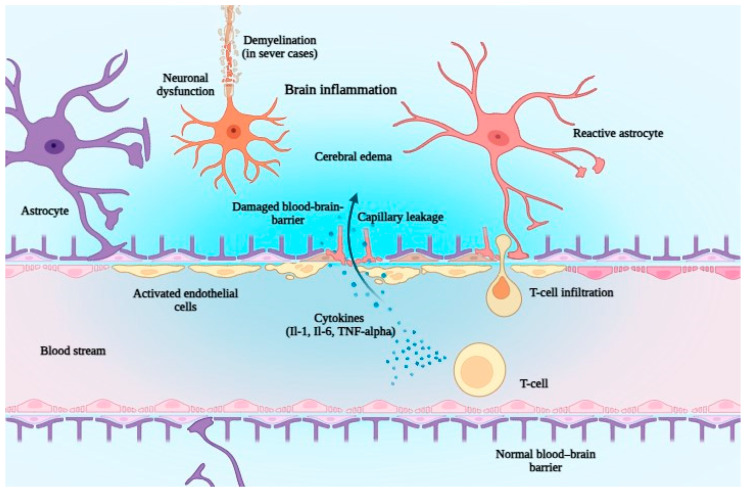
Schematic representation of the immunopathogenesis of ICANS (immune effector cell-associated neurotoxicity syndrome). Activated T-cells release inflammatory cytokines (IL-1, IL-6, and TNF-α), leading to endothelial activation, blood–brain barrier (BBB) disruption, and capillary leakage. This cascade facilitates T-cell infiltration into the CNS, triggering neuroinflammation, cerebral edema, reactive astrocytes, neuronal dysfunction, and—occasionally—demyelination in severe cases. Abbreviations: ICANS, immune effector cell-associated neurotoxicity syndrome; BBB, blood–brain barrier; IL-1, interleukin-1; IL-6, interleukin-6; TNF-α, tumor necrosis factor alpha; CNS, central nervous system. Generated with biorender.com.

Adverse events of ≥grade 3 have occurred in patients treated with CAR T-cells, with rates of 8% for CRS, 11% for neurotoxicity, and 17% for infections. CRS and neurotoxicity were significantly more frequent than in patients treated with BsAbs [45]. ICANS is believed to result from endothelial cell activation that disrupts the blood–brain barrier. This triggers an inflammatory cascade within the central nervous system, impairing both cortical and subcortical function and potentially leading to diffuse cerebral edema. The key cytokines implicated in this process include TNF-α, IL-6, and IL-1 (Figure 3) [63].

TLS is another rare but serious adverse effect associated with BsAbs therapy, occurring more frequently in the treatment of NHL. It arises from rapid BsAbs-induced tumor cell lysis, which leads to the massive release of potassium, phosphate, and nucleic acids into the bloodstream, potentially causing severe metabolic disturbances [64].

Nephrotoxicity remains underreported and insufficiently understood. The emerging evidence suggests that kidney injury may result from cytokine release, immune activation, direct renal toxicity, or underlying comorbidities [65]. Additionally, tumor lysis syndrome can contribute to acute kidney injury through electrolyte imbalances and uric acid precipitation [66].

Table 2 summarizes the adverse effects associated with BsAbs.

## 7. Conclusions and Future Developments

BsAbs represent a life-saving option for many patients with DLBCL who have a poor prognosis with conventional therapies. However, their use is associated with adverse effects and necessitates close monitoring.

BsAbs have the potential to reshape the therapeutic landscape of DLBCL, possibly altering the current sequencing of treatment lines [17]. Their integration into earlier lines of therapy is anticipated, particularly in combination with standard chemotherapy regimens [10]. Early-phase trials evaluating BsAbs in the first-line treatment of B-cell lymphomas have shown promising outcomes, raising hopes that BsAb monotherapy or BsAbs-based combinations could become standard options for DLBCL management [67].

Ongoing clinical trials are also exploring the efficacy of combining BsAbs with chemotherapy, immunotherapy, and antibody–drug conjugates (ADCs), with encouraging results in both frontline and relapsed settings [3,6].

Future research should address key questions, including the precise role of BsAbs in DLBCL treatment algorithms, the optimal therapy duration, strategies to reduce CRS risk, and mechanisms to overcome resistance [3].

## Figures and Tables

**Table 1:** Key clinical trials evaluating bispecific antibodies in diffuse large B-cell lymphoma (DLBCL).

Drug	Study Group/Target	Patients	Follow-Up	Outcomes	Reference
Epcoritamab	R/R LBCL (61.1% primary refractory, 38.9% post-CAR-T)	157	10.7 months	ORR, 63.1%; CR, 38.9%; DoR, 12 mo; CR DoR, NR.	[19]
Epcoritamab + GemOx	R/R DLBCL (transplant ineligible)	103	13.2 months	ORR, 85%; CR, 61%; CR DoR, 23.6 mo; OS, 21.6 mo.	[20]
Glofitamab	R/R B-cell NHL (incl. DLBCL)	171	Up to 27.4 months	ORR, 53.8%; CR, 36.8%. At RP2D: ORR, 65.7%; CR, 57.1%; 84.1% CRs ongoing.	[21]
Glofitamab	R/R DLBCL	154	12 months	CR, 39%; 35% post-CAR-T; median CR, 42d; 78% CRs ongoing; PFS, 37%.	[22]
Glofitamab + GemOx	R/R DLBCL (transplant ineligible)	274	20.7 months	OS: 25.5 vs. 12.9 mo (HR 0.62).	[23]
Blinatumomab	Post-auto-HSCT consolidation (DLBCL/FL)	14	1 year	86% CR at day 100; 50% CR at 1 year.	[24]
Blinatumomab	R/R DLBCL (median 3 prior therapies)	25	NR	ORR, 43%; CR, 19%; neurotoxicity with flat dosing.	[25]
Blinatumomab	Richter transformation (post-RCHOP non-CR)	25	NR	ORR, 46%; CR, 36%.	[26]
Mosunetuzumab	R/R B-NHL (aggressive and indolent)	197	NR	ORR, 34.9%/66.2%; CR, 19.4%/48.5%; CR DoR > 20 mo.	[28]
Mosunetuzumab + Polatuzumab	R/R LBCL	59	NR	ORR, 59.2%; CR, 45.9%; PFS, 11.4 mo; OS, 23.3 mo.	[29]
Mosunetuzumab + Pola-CHP	First-line DLBCL	120	24 months	CR: 72.5% vs. 77.3% (Pola-R-CHP); PFS: 70.8% vs. 81.8%.	[30]
Mosunetuzumab (post-CAR-T)	R/R LBCL post-CAR-T	30	NR	Better when given later; ↑ CD4/CD8/activated CD8 in responders.	[31]

auto-HSCT = autologous hematopoietic stem cell transplantation; B-NHL = B-cell non-Hodgkin lymphoma; CAR-T = chimeric antigen receptor T-cell therapy; CHP = cyclophosphamide, doxorubicin, and prednisone; CR = complete response; CR DoR = duration of complete response; DLBCL = diffuse large B-cell lymphoma; DoR = duration of response; FL = follicular lymphoma; GemOx = gemcitabine and oxaliplatin; HR = hazard ratio; LBCL = large B-cell lymphoma; NR = not reported; ORR = overall response rate; OS = overall survival; PFS = progression-free survival; R/R = relapsed/refractory; RCHOP = rituximab, cyclophosphamide, doxorubicin, vincristine, and prednisone; RP2D = recommended Phase 2 dose.

**Table 2:** Overview of adverse effects associated with bispecific antibody therapy.

Adverse Effect	Frequency/Severity	Mechanism/Notes
CRS	Common (up to 73.2%), grade ≥ 3: ~2%	Triggered by T-cell activation and cytokine cascade (IL-6, IL-1β, TNF-α); manageable with step-up dosing.
ICANS	Rare (~1%), lower than CAR T-cell (11%)	Related to endothelial activation and blood–brain barrier disruption; cerebral edema may occur.
Tremor	Rare, low grade (~7%)	Mild, self-limiting; less frequent than in CAR T-cell therapy.
Aphasia	Not reported with BsAbs	Frequently seen with CAR T-cells, but absent in BsAbs-treated patients.
Cytopenias	Common	Likely from cytokine-induced impaired hematopoiesis.
Febrile Neutropenia	<5%	Secondary to neutropenia.
Infections (general)	~10% grade ≥ 3	Due to lymphopenia, B/T-cell dysfunction, and hypogammaglobulinemia.
COVID-19, UTI, Pneumonia	Documented in clinical trials	Immunosuppression related.
TLS	Rare but serious	Rapid tumor breakdown → electrolyte imbalances, AKI.
AKI	Underreported, emerging concern	Cytokine effects, TLS, or comorbidities.
Hypogammaglobulinemia	Variable	Due to B-cell or plasma cell depletion (e.g., CD19/CD20/BCMA targets).
T-cell Exhaustion	Associated with infection risk	From sustained immune activation.

BsAbs—bispecific antibodies; CRS—cytokine release syndrome; ICANS—immune effector cell-associated neurotoxicity syndrome; CAR-T—chimeric antigen receptor T-cell therapy; IL-1/6—interleukin 1/6; TNF-α—tumor necrosis factor alpha; UTI—urinary tract infection; TLS—tumor lysis syndrome; AKI—acute kidney injury; BCMA—B-cell maturation antigen.

## References

[1] Barraclough A., Hawkes E.A. (2023). Antibody and Immunotherapy in Diffuse Large B-Cell Lymphoma. Semin. Hematol..

[2] D’Alò F., Bellesi S., Maiolo E., Alma E., Bellisario F., Malafronte R., Viscovo M., Campana F., Hohaus S. (2024). Novel Targets and Advanced Therapies in Diffuse Large B Cell Lymphomas. Cancers.

[3] Bennett R., Dickinson M. (2024). SOHO State of the Art Updates and Next Questions|Current Evidence and Future Directions for Bispecific Antibodies in Large B-Cell Lymphoma. Clin. Lymphoma Myeloma Leuk..

[4] Polgarova K., Trneny M. (2024). An Evaluation of Glofitamab, the First Fixed-Duration Bispecific Antibody for Relapsed or Refractory Large B-Cell Lymphomas. Expert Opin. Biol. Ther..

[5] Lu T., Zhang J., Xu-Monette Z.Y., Young K.H. (2023). The Progress of Novel Strategies on Immune-Based Therapy in Relapsed or Refractory Diffuse Large B-Cell Lymphoma. Exp. Hematol. Oncol..

[6] Saleh K., Khoury R., Khalife N., Chahine C., Ibrahim R., Tikriti Z., Le Cesne A. (2024). The Evolving Role of Bispecific Antibodies in Diffuse Large B-Cell Lymphoma. J. Pers. Med..

[7] Dogliotti I., Peri V., Clerico M., Vassallo F., Musto D., Mercadante S., Ragaini S., Botto B., Levis M., Novo M. (2024). Real Life Clinical Outcomes of Relapsed/Refractory Diffuse Large B Cell Lymphoma in the Rituximab Era: The STRIDER Study. Cancer Med..

[8] Ide D., Fujino T., Kobayashi T., Egashira A., Miyashita A., Mizuhara K., Isa R., Tsukamoto T., Mizutani S., Uchiyama H. (2024). Prognostic Model for Relapsed/Refractory Transplant-Ineligible Diffuse Large B-Cell Lymphoma Utilizing the Lymphocyte-to-Monocyte Ratio. Int. J. Hematol..

[9] Fabbri N., Mussetti A., Sureda A. (2023). Second-Line Treatment of Diffuse Large B-cell Lymphoma: Evolution of Options. Semin. Hematol..

[10] Bücklein V., Von Tresckow B., Subklewe M. (2024). T-Zell-rekrutierende Immuntherapien des B-Zell-Lymphoms—Bald in allen Therapielinien?. Dtsch. Med. Wochenschr..

[11] Moon D., Tae N., Park Y., Lee S.-W., Kim D.H. (2022). Development of Bispecific Antibody for Cancer Immunotherapy: Focus on T Cell Engaging Antibody. Immune Netw..

[12] Melody M., Gordon L.I. (2024). Sequencing of Cellular Therapy and Bispecific Antibodies for the Management of Diffuse Large B-Cell Lymphoma. Haematologica.

[13] Cosenza M., Sacchi S., Pozzi S. (2021). Cytokine Release Syndrome Associated with T-Cell-Based Therapies for Hematological Malignancies: Pathophysiology, Clinical Presentation, and Treatment. Int. J. Mol. Sci..

[14] Trabolsi A., Arumov A., Schatz J.H. (2024). Bispecific Antibodies and CAR-T Cells: Dueling Immunotherapies for Large B-Cell Lymphomas. Blood Cancer J..

[15] Frampton J.E. (2023). Epcoritamab: First Approval. Drugs.

[16] Takaura K., Ando H., Ganoza E.R. (2024). Pharmacological Characteristics and Clinical Outcomes of Epcoritamab (Recombinant) (Epkinly^®^ Subcutaneous Injection) for Malignant Lymphoma. Folia Pharmacol. Jpn..

[17] Shirouchi Y., Maruyama D. (2024). Recent Advances and Future Perspectives of T-Cell Engagers in Lymphoid Malignancies. Jpn. J. Clin. Oncol..

[18] Minson A.G., Dickinson M.J. (2025). New Bispecific Antibodies in Diffuse Large B-Cell Lymphoma. Haematologica.

[19] Thieblemont C., Karimi Y.H., Ghesquieres H., Cheah C.Y., Clausen M.R., Cunningham D., Jurczak W., Do Y.R., Gasiorowski R., Lewis D.J. (2024). Epcoritamab in Relapsed/Refractory Large B-Cell Lymphoma: 2-Year Follow-up from the Pivotal EPCORE NHL-1 Trial. Leukemia.

[20] Brody J.D., Jørgensen J., Belada D., Costello R., Trněný M., Vitolo U., Lewis D.J., Karimi Y.H., Sureda A., André M. (2025). Epcoritamab plus GemOx in Transplant-Ineligible Relapsed/Refractory DLBCL: Results from the EPCORE NHL-2 Trial. Blood.

[21] Hutchings M., Morschhauser F., Iacoboni G., Carlo-Stella C., Offner F.C., Sureda A., Salles G., Martínez-Lopez J., Crump M., Thomas D.N. (2021). Glofitamab, a Novel, Bivalent CD20-Targeting T-Cell–Engaging Bispecific Antibody, Induces Durable Complete Remissions in Relapsed or Refractory B-Cell Lymphoma: A Phase I Trial. J. Clin. Oncol..

[22] Dickinson M.J., Carlo-Stella C., Morschhauser F., Bachy E., Corradini P., Iacoboni G., Khan C., Wróbel T., Offner F., Trněný M. (2022). Glofitamab for Relapsed or Refractory Diffuse Large B-Cell Lymphoma. N. Engl. J. Med..

[23] Abramson J.S., Ku M., Hertzberg M., Huang H.-Q., Fox C.P., Zhang H., Yoon D.H., Kim W.-S., Abdulhaq H., Townsend W. (2024). Glofitamab plus Gemcitabine and Oxaliplatin (GemOx) versus Rituximab-GemOx for Relapsed or Refractory Diffuse Large B-Cell Lymphoma (STARGLO): A Global Phase 3, Randomised, Open-Label Trial. Lancet.

[24] Ghobadi A., Foley N.C., Cohen J., Rettig M.P., Cashen A.F., Gehrs L., Christ S., Street E., Wallace N., Ritchey J. (2024). Blinatumomab Consolidation Post–Autologous Stem Cell Transplantation in Patients with Diffuse Large B-Cell Lymphoma. Blood Adv..

[25] Viardot A., Goebeler M.-E., Hess G., Neumann S., Pfreundschuh M., Adrian N., Zettl F., Libicher M., Sayehli C., Stieglmaier J. (2016). Phase 2 Study of the Bispecific T-Cell Engager (BiTE) Antibody Blinatumomab in Relapsed/Refractory Diffuse Large B-Cell Lymphoma. Blood.

[26] Guièze R., Ysebaert L., Roos-Weil D., Fornecker L.-M., Ferrant E., Molina L., Aurran T., Clavert A., De Guibert S., Michallet A.-S. (2024). Blinatumomab after R-CHOP Bridging Therapy for Patients with Richter Transformation: A Phase 2 Multicentre Trial. Nat. Commun..

[27] Matasar M., Bartlett N.L., Shadman M., Budde L.E., Flinn I., Gregory G.P., Kim W.S., Hess G., El-Sharkawi D., Diefenbach C.S. (2024). Mosunetuzumab Safety Profile in Patients with Relapsed/Refractory B-Cell Non-Hodgkin Lymphoma: Clinical Management Experience from a Pivotal Phase I/II Trial. Clin. Lymphoma Myeloma Leuk..

[28] Budde L.E., Assouline S., Sehn L.H., Schuster S.J., Yoon S.-S., Yoon D.H., Matasar M.J., Bosch F., Kim W.S., Nastoupil L.J. (2022). Single-Agent Mosunetuzumab Shows Durable Complete Responses in Patients with Relapsed or Refractory B-Cell Lymphomas: Phase I Dose-Escalation Study. J. Clin. Oncol..

[29] Budde L.E., Olszewski A.J., Assouline S., Lossos I.S., Diefenbach C., Kamdar M., Ghosh N., Modi D., Sabry W., Naik S. (2024). Mosunetuzumab with Polatuzumab Vedotin in Relapsed or Refractory Aggressive Large B Cell Lymphoma: A Phase 1b/2 Trial. Nat. Med..

[30] Westin J., Phillips T.J., Mehta A., Hoffmann M.S., Gonzalez-Barca E., Thieblemont C., Bastos-Oreiro M., Greil R., Giebel S., Wei M.C. (2025). Mosunetuzumab plus Pola-CHP Compared with Pola-R-CHP in Previously Untreated DLBCL: Final Results from a Phase 2 Study. Blood Adv..

[31] Chong E.A., Penuel E., Napier E.B., Lundberg R.K., Budde L.E., Shadman M., Matasar M.J., Bartlett N.L., Flinn I.W., Bosch F. (2025). Impact of Prior CAR T-Cell Therapy on Mosunetuzumab Efficacy in Patients with Relapsed or Refractory B-Cell Lymphomas. Blood Adv..

[32] Shahzad M., Khalid M.F., Amin M.K., Basharat A., Ammad-Ud-Din M., Park R., Anwar I., Faisal M.S., Jaglal M. (2024). Geographic and Racial Disparities in Chimeric Antigen Receptor–T Cells and Bispecific Antibodies Trials Access for Diffuse Large B-Cell Lymphoma. Clin. Lymphoma Myeloma Leuk..

[33] Davila M.L., Brentjens R., Wang X., Rivière I., Sadelain M. (2012). How Do CARs Work?: Early Insights from Recent Clinical Studies Targeting CD19. OncoImmunology.

[34] Sadelain M., Brentjens R., Rivière I. (2013). The Basic Principles of Chimeric Antigen Receptor Design. Cancer Discov..

[35] (2017). First-Ever CAR T-Cell Therapy Approved in U.S. Cancer Discov..

[36] Si Lim S.J., Grupp S.A., DiNofia A.M. (2021). Tisagenlecleucel for Treatment of Children and Young Adults with Relapsed/Refractory B-cell Acute Lymphoblastic Leukemia. Pediatr. Blood Cancer.

[37] Fowler N.H., Dickinson M., Dreyling M., Martinez-Lopez J., Kolstad A., Butler J. (2022). Tisagenlecleucel Is Safe and Effective in Relapsed/Refractory Follicular Lymphoma. Cancer Discov..

[38] Bouchkouj N., Kasamon Y.L., De Claro R.A., George B., Lin X., Lee S., Blumenthal G.M., Bryan W., McKee A.E., Pazdur R. (2019). FDA Approval Summary: Axicabtagene Ciloleucel for Relapsed or Refractory Large B-Cell Lymphoma. Clin. Cancer Res..

[39] Bouchkouj N., Zimmerman M., Kasamon Y.L., Wang C., Dai T., Xu Z., Wang X., Theoret M., Purohit-Sheth T., George B. (2022). FDA Approval Summary: Axicabtagene Ciloleucel for Relapsed or Refractory Follicular Lymphoma. Oncol..

[40] De Ramon Ortiz C., Wang S., Stathis A., Bertoni F., Zenz T., Novak U., Simonetta F. (2024). How to Integrate CD19 Specific Chimeric Antigen Receptor T Cells with Other CD19 Targeting Agents in Diffuse Large B-cell Lymphoma?. Hematol. Oncol..

[41] Valery M., Saleh K., Ecea R., Michot J.M., Ribrag V., Fizazi K., Hollebecque A., Lecesne A., Ponce S., Loriot Y. (2023). Infections Occurring Following IL6 Blockade for the Management of Cytokine Release Syndrome in Onco-Hematology Patients. Cancer Chemother. Pharmacol..

[42] Tumuluru S., Godfrey J.K., Cooper A., Yu J., Chen X., MacNabb B.W., Venkataraman G., Zha Y., Pelzer B., Song J. (2024). Integrative Genomic Analysis Identifies Unique Immune Environments Associated with Immunotherapy Response in Diffuse Large B Cell Lymphoma. bioRxiv.

[43] Brooks T.R., Caimi P.F. (2024). A Paradox of Choice: Sequencing Therapy in Relapsed/Refractory Diffuse Large B-Cell Lymphoma. Blood Rev..

[44] Park J.H., Nath K., Devlin S.M., Sauter C.S., Palomba M.L., Shah G., Dahi P., Lin R.J., Scordo M., Perales M.-A. (2023). CD19 CAR T-Cell Therapy and Prophylactic Anakinra in Relapsed or Refractory Lymphoma: Phase 2 Trial Interim Results. Nat. Med..

[45] Kim J., Cho J., Lee M.H., Yoon S.E., Kim W.S., Kim S.J. (2024). CAR T Cells vs Bispecific Antibody as Third- or Later-Line Large B-Cell Lymphoma Therapy: A Meta-Analysis. Blood.

[46] Topp M.S., Matasar M., Allan J.N., Ansell S.M., Barnes J.A., Arnason J.E., Michot J.-M., Goldschmidt N., O’Brien S.M., Abadi U. (2025). Odronextamab Monotherapy in R/R DLBCL after Progression with CAR T-Cell Therapy: Primary Analysis of the ELM-1 Study. Blood.

[47] Sousa-Pimenta M., Martins Â., Mariz J.M., Berraondo P. (2023). Response to Therapy in Richter Syndrome: A Systematic Review with Meta-Analysis of Early Clinical Trials. Front. Immunol..

[48] Carlo-Stella C. (2024). Relapse after Glofitamab, a Novel Unmet Medical Need with High Rates of CD20 Loss. Br. J. Haematol..

[49] Burt R., Warcel D., Fielding A.K. (2019). Blinatumomab, a Bispecific B-Cell and T-Cell Engaging Antibody, in the Treatment of B-Cell Malignancies. Hum. Vaccines Immunother..

[50] Gaballa S., Hou J.-Z., Devata S., Cho S.-G., Nair R., Yoon D.H., Jacobs R., Izutsu K., Stevens D.A., Maruyama D. (2024). Evaluation of AZD0486, a Novel CD19xCD3 T-Cell Engager, in Relapsed/Refractory Diffuse Large B-Cell Lymphoma in an Ongoing First-in-Human Phase 1 Study: High Complete Responses Seen in CAR-T-Naive and CAR-T-Exposed Patients. Blood.

[51] Leclercq-Cohen G., Steinhoff N., Albertí Servera L., Nassiri S., Danilin S., Piccione E., Yángüez E., Hüsser T., Herter S., Schmeing S. (2023). Dissecting the Mechanisms Underlying the Cytokine Release Syndrome (CRS) Mediated by T-Cell Bispecific Antibodies. Clin. Cancer Res..

[52] Moreau P., Touzeau C. (2022). T-Cell–Redirecting Bispecific Antibodies in Multiple Myeloma: A Revolution?. Blood.

[53] Juluri K.R., Wu Q.V., Voutsinas J., Hou J., Hirayama A.V., Mullane E., Miles N., Maloney D.G., Turtle C.J., Bar M. (2022). Severe Cytokine Release Syndrome Is Associated with Hematologic Toxicity Following CD19 CAR T-Cell Therapy. Blood Adv..

[54] Bannerji R., Arnason J.E., Advani R.H., Brown J.R., Allan J.N., Ansell S.M., Barnes J.A., O’Brien S.M., Chávez J.C., Duell J. (2022). Odronextamab, a Human CD20×CD3 Bispecific Antibody in Patients with CD20-Positive B-Cell Malignancies (ELM-1): Results from the Relapsed or Refractory Non-Hodgkin Lymphoma Cohort in a Single-Arm, Multicentre, Phase 1 Trial. Lancet Haematol..

[55] Gurumurthi A., Westin J., Subklewe M. (2023). The Race Is on: Bispecifics vs CAR T Cells in B-Cell Lymphoma. Blood Adv..

[56] Budde L.E., Sehn L.H., Matasar M., Schuster S.J., Assouline S., Giri P., Kuruvilla J., Canales M., Dietrich S., Fay K. (2022). Safety and Efficacy of Mosunetuzumab, a Bispecific Antibody, in Patients with Relapsed or Refractory Follicular Lymphoma: A Single-Arm, Multicentre, Phase 2 Study. Lancet Oncol..

[57] Morschhauser F., Dahiya S., Palomba M.L., Martin Garcia-Sancho A., Reguera Ortega J.L., Kuruvilla J., Jäger U., Cartron G., Izutsu K., Dreyling M. (2024). Lisocabtagene Maraleucel in Follicular Lymphoma: The Phase 2 TRANSCEND FL Study. Nat. Med..

[58] Falchi L., Hutchings M., Carlo-Stella C., Morschhauser F., Dickinson M., Cartron G., Khan C., Tani M., Martinez-Lopez J., Bartlett N.L. (2024). Dexamethasone Is Associated with Reduced Frequency and Intensity of Cytokine Release Syndrome Compared with Alternative Corticosteroid Regimens as Premedication for Glofitamab in Patients with Relapsed/Refractory Large B-Cell Lymphoma. Haematologica.

[59] Izutsu K., Kumode T., Yuda J., Nagai H., Mishima Y., Suehiro Y., Yamamoto K., Fujisaki T., Ishitsuka K., Ishizawa K. (2023). Subcutaneous Epcoritamab Monotherapy in Japanese Adults with Relapsed/Refractory Diffuse Large B-cell Lymphoma. Cancer Sci..

[60] Thieblemont C., Phillips T., Ghesquieres H., Cheah C.Y., Clausen M.R., Cunningham D., Do Y.R., Feldman T., Gasiorowski R., Jurczak W. (2023). Epcoritamab, a Novel, Subcutaneous CD3xCD20 Bispecific T-Cell–Engaging Antibody, in Relapsed or Refractory Large B-Cell Lymphoma: Dose Expansion in a Phase I/II Trial. J. Clin. Oncol..

[61] Kim W.-S., Kim T.M., Cho S.-G., Jarque I., Iskierka-Jażdżewska E., Poon M.L., Prince H.M., Oh S.Y., Lim F., Carpio C. (2022). Odronextamab in Patients with Relapsed/Refractory (R/R) Diffuse Large B-Cell Lymphoma (DLBCL): Results from a Prespecified Analysis of the Pivotal Phase II Study ELM-2. Blood.

[62] Van De Donk N.W.C.J., Zweegman S. (2023). T-Cell-Engaging Bispecific Antibodies in Cancer. Lancet.

[63] Danish H., Santomasso B.D. (2021). Neurotoxicity Biology and Management. Cancer J..

[64] Omer M.H., Shafqat A., Ahmad O., Alkattan K., Yaqinuddin A., Damlaj M. (2023). Bispecific Antibodies in Hematological Malignancies: A Scoping Review. Cancers.

[65] Wen X., Xu G. (2024). Nephrotoxicity in Bispecific Antibodies Recipients: Focus on T-Cell-Engaging Bispecific Antibodies. OncoTargets Ther..

[66] Uckun F.M., Lin T.L., Mims A.S., Patel P., Lee C., Shahidzadeh A., Shami P.J., Cull E., Cogle C.R., Watts J. (2021). A Clinical Phase 1B Study of the CD3xCD123 Bispecific Antibody APVO436 in Patients with Relapsed/Refractory Acute Myeloid Leukemia or Myelodysplastic Syndrome. Cancers.

[67] Sun H., Xing H., Han L., Song Y., Jiang Z., Liu Y., Yu J. (2024). Bispecific Antibodies Targeting CD20xCD3 in Immunotherapy for Adult B-Cell Lymphoma: Insights from the 65th American Society of Hematology 2023 Annual Meeting. Expert Opin. Biol. Ther..

